# Norwegian General Practitioners’ Perspectives on Implementation of a Guided Web-Based Cognitive Behavioral Therapy for Depression: A Qualitative Study

**DOI:** 10.2196/jmir.3556

**Published:** 2014-09-10

**Authors:** Maja Wilhelmsen, Ragnhild Sørensen Høifødt, Nils Kolstrup, Knut Waterloo, Martin Eisemann, Richard Chenhall, Mette Bech Risør

**Affiliations:** ^1^Department of Community MedicineFaculty of Health SciencesUiT The Arctic University of NorwayTromsøNorway; ^2^Department of PsychologyFaculty of Health SciencesUiT The Arctic University of NorwayTromsøNorway; ^3^Center for Health EquitySchool of Population and Global HealthUniversity of MelbourneMelbourneAustralia

**Keywords:** mental health, Internet, telemedicine, qualitative research, primary health care, cognitive therapy, depression

## Abstract

**Background:**

Previous research suggests that Internet-based cognitive behavioral therapy (ICBT) has a positive effect on symptoms of depression. ICBT appears to be more effective with therapist support, but it is unclear what this support should comprise. General practitioners (GPs) have positive attitudes toward ICBT. However, ICBT is rarely used in regular care in general practice. More research is warranted to integrate the potential of ICBT as part of regular care.

**Objective:**

The aim of this study was to explore aspects perceived by GPs to affect the implementation of guided ICBT in daily practice. Understanding their perspectives may contribute to improving the treatment of depression in the context of general practice.

**Methods:**

A training package (3-day course) introducing a Norwegian translation of the ICBT program MoodGYM was developed and presented to GPs in Norway. Following training, GPs were asked to include guided ICBT in their regular care of patients with symptoms of depression by providing brief, face-to-face follow-up consultations between modules. We interviewed 11 GPs who had taken the course. Our interview guide comprised open questions that encouraged GPs to frame their responses using examples from their experiences when implementing ICBT. Thematic analysis was chosen to explore patterns across the data.

**Results:**

An overall belief that ICBT would benefit both the patients’ health and the GPs’ own work satisfaction prompted the GPs to take the ICBT course. ICBT motivated them to invest time and effort in improving treatment. The most important motivating aspects in MoodGYM were that a program based on cognitive behavioral therapy could add a structured agenda to their consultations and empower depressed patients. Organizational aspects, such as a lack of time and varied practice, inhibited the use of ICBT. Inadequate knowledge, recalling the program, and changing own habits were also challenging. The GPs were ambivalent about whether ICBT had a negative impact on the doctor–patient interaction in the module follow-ups. Generally, GPs made an effort to recommend MoodGYM, but the expected module follow-ups were often not provided to patients and instead the GPs returned to standard treatment.

**Conclusions:**

GPs’ feedback in the present study contribute to our understanding of the challenges of changing treatment for depression. Our findings indicated that recommending ICBT could add to the GP’s toolkit. Offering training and highlighting the following aspects may increase recommendation of ICBT by GPs: (1) ICBT is theory-based and credible, (2) ICBT increases the GPs’ work satisfaction by having a tool to offer, and (3) ICBT facilitates empowerment of patients in their own health. In addition, the present study also indicated that complex aspects must be accommodated before module follow-ups can be incorporated into GPs’ treatment of depression.

## Introduction

### Overview

Every year, it is estimated that more than one-third of the European population suffers from mental disorders, with depression and anxiety being the most frequent [1,2]. Depression imposes tremendous emotional, financial, and social burdens for patients, their families, and society [3]. Mental disorders have been suggested as one of the biggest health challenges because of deficiencies in available treatment and poor service provision. Rethinking our provision of mental health care is needed [1,4]. Mental health patients tend to prefer consultations with a therapist to prescribed medication [5,6]. Standard cognitive behavioral therapy (CBT) in consultations in routine specialized mental health services is effective but time consuming [7], making this therapy inaccessible to many.

### Internet-Based Treatment of Depression

In multiple trials, the use of Internet-based CBT (ICBT) has shown promising results in treating depression (eg, [8-11]). ICBT can be self-administered or supported by either minimal-contact follow-ups or by guided follow-ups that focus on process issues. It is unclear what the support should comprise [12]. However, guided ICBT appears to be more effective than self-administered ICBT [8,9,13]. Guided ICBT may also be as effective as standard face-to-face therapy [9,14]. Furthermore, patients value being active agents using ICBT, although they also emphasize the helpfulness of a relationship with a trusted clinician [15]. In Norway, an increasing proportion of the population uses the Internet for health purposes, and in 2010 the proportion was 77% [16]. This may indicate that the population is amenable to supported online supplements, such as guided ICBT.

### Treatment of Depression in Primary Care

Research has recommended that mental illness should be detected and treated early, before more severe expressions can occur [1,17]. GPs are often the first point of professional contact for individuals with depression, and most of these patients are currently treated in primary care [1,18]. In Norway, the mental health provider in primary care is most often the GP [19]. In guidelines from the National Institute for Health and Clinical Excellence [20] and in Norwegian national guidelines for treatment of depression [19], it is recommended initially to apply low-intensity, non-pharmacological approaches such as CBT-based self-help or online interventions in the treatment of mild to moderate depression. Studies have found that GPs have positive attitudes toward such interventions [5,21,22]. However, other studies have shown that GPs rarely recommend evidence-based self-management or eHealth programs to patients with depressive symptoms [18,22].

ICBT has been suggested to be effective in primary care even if the provider (ie, the GP) lacks extensive specialized training [6,9,23-25]. MoodGYM is an ICBT program developed at the Centre for Mental Health Research at the Australian National University. It has been proven in trials to be effective in alleviating depression as a self-administered self-help program [26] and as guided ICBT for patients from primary care [10]. However, there is a gap in the research evidence related to the conclusions of trials and the knowledge about how to implement new methods in regular care [27,28]. More research is needed to reduce this gap and to explore the potential of ICBT deployed in everyday clinical settings [21,23]. Little is known about trained GPs’ experiences of implementing guided ICBT into regular care.

A wealth of theories have been developed to explain aspects affecting the implementation of innovations in health care [29]. We chose normalization process theory (NPT), developed by May and Finch [27,30], as a framework when investigating implementation of ICBT because NPT is derived from multiple qualitative studies exploring the implementation of complex interventions and eHealth contextualized in regular health practice. NPT suggests that, for the health professional, successful implementation depends on a complex interplay of the following four main components of “work”: (1) coherence, whereby the participants make sense of an intervention, (2) cognitive participation, which requires the participants to engage in the intervention, (3) collective action, which requires that efforts are made to enable the intervention to happen, and (4) reflexive monitoring, comprising formal and informal appraisals of the benefits and costs of the intervention. Small improvements in the treatment of depression in general practice can have positive consequences for many patients. The aim of this study was to explore those aspects perceived by GPs to affect their implementation of guided ICBT in daily practice. Understanding their perspectives may contribute to improving the treatment of depression in the context of general practice.

## Methods

### Study Context

**Figure 1:**
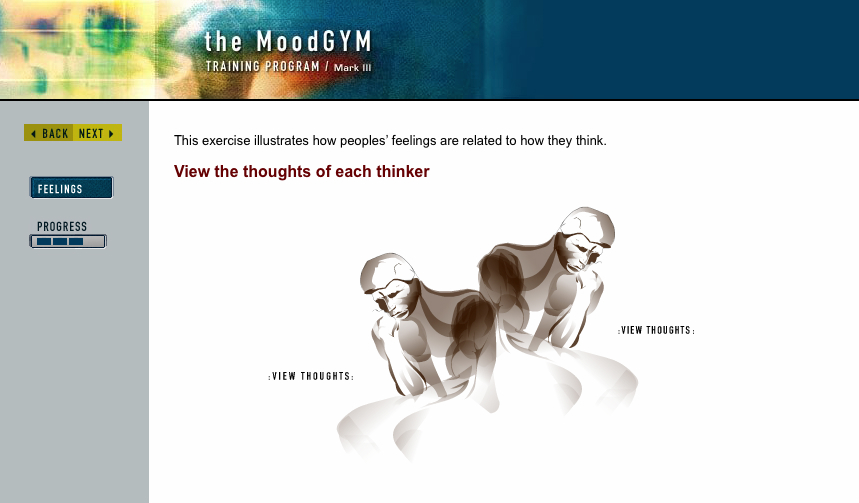
Sample screenshot from MoodGYM.

A training package based on a Norwegian translation of the ICBT program MoodGYM was developed and presented by a GP (NK) and two psychologists (RSH and Kjersti Lillevoll) as a 3-day course for GPs in spring 2011. MoodGYM is a free Internet-based self-help program comprising five interactive modules introducing cognitive behavioral principles through online exercises. MoodGYM demonstrates the relationship between thoughts and emotions and teaches relaxation techniques. It also includes sections on managing relationships and increasing engagement in positive activities. A sample screenshot from MoodGYM is presented in Figure 1. The course consisted of an introduction to CBT principles, presentation of and a group session on MoodGYM’s content, and presentation of a manual for follow-ups. The manual was also supplied online and contained a short summary of each module and suggestions for follow-up questions. In the final session, a patient described his experiences with guided ICBT.

**Figure 2:**
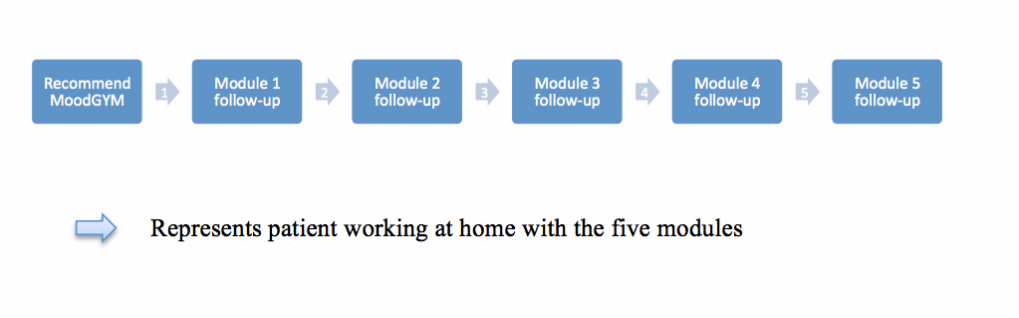
ICBT with module follow-ups.

The course recommended guided ICBT as follows: (1) to recommend MoodGYM to patients with symptoms of depression and subsequently (2) to provide module follow-ups comprising brief and structured face-to-face consultations between the online modules (see Figure 2). The follow-ups were meant to be of a motivating nature, by offering a dialogue on process issues and allowing some time for reflection. Process issues were related to working with and understanding principles presented in the online modules such as what was difficult and what was useful. Follow-ups were suggested as 20-minute consultations every second week. GPs who currently worked in general practice and who completed the course all signed up for further research on implementation of the guided ICBT.

### Participants

A purposive sample [31] of 11 GPs from northern Norway was included, and interview arrangements were made over the telephone. The study originally included GPs who had enrolled voluntarily in the guided ICBT course. In the process of recruiting, we had the opportunity to include two additional GPs who had attended a 3-hour presentation of this treatment model given by one of the GPs who had attended the course. These two GPs were included in the analysis primarily for comparative purposes, to add an extra dimension to the analysis of the GPs’ experiences with ICBT. Participants were both men and women, of various ages, and with various lengths of experience as GPs (Table 1).

**Table 1:** Participant characteristics.

GP	Age	Date of Interview	Duration of interview, minutes	Experience as a GP, years	Recommend MoodGYM	Gender	3-day course	Interview venue
1	52	06.02.13	40	21	yes	male	yes	office
2	35	14.02.13	50	3.5	yes	female	yes	home
3	53	20.02.13	33	21	yes	male	no	office
4	33	25.02.13	35	3.5	yes	female	yes	home
5	38	25.05.13	47	8	yes	female	yes	home
6	58	12.03.13	70	28	no	female	yes	office
7	42	14.03.13	85	3	yes	female	yes	office
8	54	20.03.13	75	10	yes	female	yes	university
9	45	08.04.13	76	12	yes	female	yes	office
10	51	12.09.13	60	12	yes	female	no	office
11	34	19.09.13	83	3.5	yes	female	yes	office

### Interview Schedule and Data Collection

The female interviewers (MW and RSH) conducted semistructured in-depth interviews designed to gather information about (1) the GPs’ general views on their work with depressed patients, (2) motivational aspects for learning ICBT, (3) experiences from implementing guided ICBT, and (4) implications of the use of ICBT for consultation quality and patient-doctor interaction. The interview guide (see Multimedia Appendix 1) was developed by the interdisciplinary author group combining prior experience within eHealth research, clinical experience from general practice, and long experience from medical anthropology. A pilot was not conducted; however, adjustments were made after three interviews. The venue was flexible, and GPs chose their home, office, or the University of Tromsø. Individual interviews lasted 33-85 minutes and were audio recorded. The interviews were conducted as face-to-face dialogues, and open-ended questions were used to evoke descriptions of situations grounded contextually in everyday events from their clinical practice. The emphasis was on working together in the interview to reach an understanding of the GPs’ experiences that reflected their perceptions and attitudes [31]. The GPs were not invited to give feedback later in the research process. Field notes were recorded immediately after interviews.

### Data Analysis

Interviews were transcribed into NVivo 10 data analysis software and anonymized. Thematic analysis is an accessible stepwise method often used to investigate patterns across datasets [32]. It is also a flexible method compatible with an inductive, data-driven approach. We chose thematic analysis as a method because it is suitable when exploring patterns in stories that describe experiences and meanings. Our aim was to explore all aspects perceived by the GPs to have affected their implementation of ICBT, and thus, an inductive approach was chosen. The initial analysis involved repeated readings of each transcript to obtain an overall impression. The next phase involved coding the entire dataset on a semantic level. Events, thoughts, and actions were coded as themes on the basis of their ability to capture content with meanings that were important in relation to the overall research question. Subsequently, we identified and interpreted overarching themes in a constant process of moving between the data, potential themes, and maps made for visualization. A storyline extending from what initially attracted the GPs to take the course to how they applied ICBT in the everyday clinical setting was identified. We worked toward elucidating themes that were internally homogeneous and externally heterogeneous and had explanatory power. Finally, the themes were considered if they were coherent and represented the meanings found in the interviews [32]. Throughout the analytical process, all findings were discussed and validated with an experienced qualitative researcher (MBR) and two GPs (MW and NK). Inconsistencies were resolved through discussion and further reflection. All transcripts have been checked against the audio recordings. Inspired by NPT [27,30], the researchers were sensitized to components in interplay that are suggested as important during implementation of the complex interventions. Further, findings were discussed in light of existing literature.

### Ethics Approval

Written informed consent was obtained from all participants. Ethical approval was given by the Regional Ethical Committee, Tromsø 2011/2163.

## Results

### Key Themes

We identified four key themes and present here the common storyline extending from what initially attracted the GPs to take the course to how they applied ICBT in the everyday clinical setting. The four key themes are: “GPs wanted to improve treatment and work satisfaction”, “The value of a structured agenda and the patient as an active agent in treatment”, “Constraints of hectic practice, inadequate knowledge, and competing tasks”, and “GPs recommended it, but few module follow-ups were provided”.

### GPs Wanted to Improve Treatment and Work Satisfaction

GPs reflected on what initially attracted them to take the course and how they could make sense of a change in the treatment of depression they normally provided, in the following referred to as standard treatment. GPs noted that symptoms of depression were common and treatment was considered part of their job. They had inadequate access to specialist care, and thus referral was often difficult. GPs hoped that implementation of an online intervention would benefit patients with mental health issues. Comments that implied a need to improve GPs’ own treatment were prominent:

[We give] what we call supporting consultations in general practice, but actually it is just talking without any…I wouldn’t say meaning, but no concrete agenda in a way. I mean, there is no…It is just: How is it going? What have you been doing lately? It is more the patient talking in a free way about how they are and stuff. But no actual therapy really.

Participant 11

Many times I feel that I don’t have an agenda or further program other than these supportive consultations, which I often feel it [depression treatment] ends up with.

Participant 5

GPs used devaluating words such as “only supportive consultations” or “just talking” when they referred to standard treatment, and some described the situation with frustration, indicating concerns about the quality of care. They hoped that supplementing depression treatment would result in improved health and quicker recovery for their patients.

Standard treatment was described as self-taught, informal, and unstructured. The frustration identified might also imply a sense of lack of control and vulnerability. Others expressed that their treatment had improved with experience and was helpful, but they felt they needed something more to offer their patients: “And I think many can feel the frustration about what should we do. How can we help these patients [with depression]? In what way?...To me it felt very good [to learn ICBT]! Because now I finally felt I had some treatment I could try” [Participant 11].

The word “tool” was often used to describe how implementing ICBT could supplement treatment. This indicated another important aspect of the GPs’ reasons for enrollment; that is, they wanted to improve their work satisfaction by increasing their competence and confidence by adding a concrete instrument to their treatment of depression: “So it is, it is nice to have a tool to offer people. That in itself makes you feel better as a caregiver. You feel more professional!” [Participant 1].

According to NPT, coherence is experienced if the participants find an intervention to be a good idea and they are able to define the intervention [27,30]. The GPs showed coherence as they found recommending an online intervention to be a good idea and felt that competence in delivering this type of intervention would be helpful for the patient and of benefit to the GPs. When they described the use of MoodGYM, they talked about it as a supplemental tool, but they did not explicitly define what module follow-ups should comprise and why module follow-ups would improve treatment. This might indicate limited coherence for the module follow-ups.

### The Value of a Structured Agenda and the Patient as an Active Agent in Treatment

Internet self-help was regarded as a good idea. The GPs commented further on what aspects of MoodGYM engaged them, enabling them to invest time and effort. Overall, the GPs valued that MoodGYM added a structured agenda to treatment in two ways. First, GPs described MoodGYM’s educational content enthusiastically because it is systematic and based on CBT and therefore added a theoretical basis to the treatment: “I find it [MoodGYM] to be…it is very systematic and built up in a good way with the different modules...Yes, I think it is really great and it helps…it is a good help for me as a GP to have something to recommend so that the patients can be educated” [Participant 7] and “To in a way get theory in and...That you [patient] have an opportunity to go into a webpage where there are concrete tasks. I found that positive” [Participant 11].

GPs had confidence in the developers and felt more skillful when able to recommend an evidence-based Web intervention, which implied they felt more professional in their ability to address depression. Compared with the others, the two GPs (Participants 3 and 10) who had not taken the course but had only been to a presentation were more reluctant to tell patients and colleagues that they acknowledged the content as credible and appreciated the structure of ICBT. This may indicate that the course strengthened confidence and the perceived credibility of MoodGYM.

GPs were familiar with referring to the Internet for other conditions. Convenience was mentioned as a positive aspect of recommending MoodGYM because it is free and available, which means it can fit easily into depressed patients’ lives. GPs commented that they could help solve their patients’ mental health issues only through the efforts of the patients themselves. Patients were regarded as their own best helpers, and the GPs found that ICBT could facilitate self-help. This implied a second aspect of what the GPs found valuable: viewing MoodGYM as a structured agenda to empower patients as active agents to contribute to their own health and recovery. “You give the responsibility to the patient, the responsibility to become better. You place the responsibility. It doesn’t work like [the patient can] just come to me and be cured of his depression. He [the patient] needs to do it himself” [Participant 4].

Evaluation was needed to find patients suitable for ICBT, and different characteristics were considered to contribute positively to the success of ICBT. Patients who accepted that their problem was psychological and who could reflect on their own condition were considered suitable for ICBT. A young age was considered positive because young people were assumed to be more computer literate and because the style of MoodGYM appealed to young people. Motivation and initiative were essential: “The people [who] I think will be able to make MoodGYM useful are those who are self-motivated. Those who really want it. And…I mean, they need to have some determination in them. Then I think it will be useful” [Participant 4].

The severity of depression also had to be considered because depression by itself has negative influences on initiative, concentration, and motivation. GPs considered the program best suited to people with mild to moderate depression.

Theory-based structured agenda in the treatment and empowerment of patients were the most important aspects to promote engagement. This engagement relates to the NPT component of cognitive participation [27,30].

### Constraints of Hectic Practice, Inadequate Knowledge, and Competing Tasks

Engaging aspects of the program were reported; however, GPs also discussed several challenges in using ICBT. One challenge mentioned was patients being negative to ICBT or not adhering. In the organizational context of general practice, lack of time in the consultation was also a recurrent constraint. The structure of a typical day comprised a tight appointment schedule, no time for preparation between patients, and 20-minute consultations. GPs commented that this made it difficult to take the time needed to think about new treatment methods and remember to recommend MoodGYM to patients: “You already have so little time in the consultation in general practice that everything has to be ready when the patient comes in…It is just opening the book to check what they are coming for. And then things must be ready…It [guided ICBT] isn’t done in 20 minutes” [Participant 5].

Subsequent to recommending MoodGYM, the GPs were asked to provide module follow-ups; however, they described this as a difficult task. Lack of time worsened as a patient often has several issues to discuss in follow-up consultations. Given that the GPs saw a variety of patients, it could also be a long time between each encounter with a suitable patient. The lack of continuity made it difficult for the GPs to become familiar with and knowledgeable about MoodGYM, and thus it was demanding to change their habits. Inadequate knowledge about the modules and prioritizing time to go through modules themselves was a challenge because of other competing tasks and lack of incitement to do so. A need for detailed knowledge indicated that they had high standards for the competence needed to understand and apply MoodGYM’s content and to talk about the modules with their patients. Lack of practical training of module follow-ups in the course was noted as an element that made it difficult to integrate it to a clinical setting. The hectic day also made it difficult to find and apply the manual for module follow-ups: “I should have done it all [entire MoodGYM] so I would know what they [the patients] have been through each time…But I haven’t had the time yet” [Participant 4] and “You had made such nice manuals for each module…I felt it was a mess in my things…And it is about me as well, but I think I should have known it [each module] better!” [Participant 5].

Ambivalent findings were identified on how module follow-ups might affect the interaction with the patients. Dialogue about the programs’ content could give a common platform to talk about. On the other hand, a focus on process issues from working with MoodGYM was thought to inhibit other tasks perceived to be important in standard treatment, such as the open dialogue:

I find it a barrier [to give module follow-ups]…Yes, it breaks in a way with how you normally work in a way. So I think that is one of the main reasons for not.

Participant 9

Interviewer: What kinds of elements are most important in the treatment you give?

No, it must be to listen to the patients…They must feel safe…must be safe to meet me. I think that is the most important to have a relationship. And you must have a relationship to get a dialogue, which enables reflection of the patients themselves…The fact that they get a recommendation to MoodGYM and that, it doesn’t influence the actual conversation [in further consultations].

Participant 9

Overall, many inhibiting aspects on engagement were identified: limited time, lack of detailed knowledge about the modules, the variety of patients seen in the practice, and competing tasks in the consultation. These constraints applied strongly to the module follow-ups. The value of discussing process issues in the consultations was considered problematic. This may indicate little cognitive participation similar to limited coherence [27,30] in this part of the treatment.

### GPs Recommended It, But Few Module Follow-Ups Were Provided

All GPs tried to implement MoodGYM as best as they could remember and apply it within a hectic day. Many had the Internet address ready to give to patients. To various extents, they found suitable patients and took the time to recommend MoodGYM. One exception was a GP (Participant 6) who chose not to recommend MoodGYM and explained that it was too demanding to change her treatment habits, although she felt that this would have been different if she had been younger. Other feedback included “If I were to estimate [how many patients I have recommended it to] I would think 25” [Participant 1] and “And I have recommended it to some patients. Especially young people who are on the Internet all the time” [Participant 3].

GPs applied strategies to promote the use of MoodGYM. Motivation was essential and to increase patients’ motivation, GPs emphasized the importance of recommending it in a convincing way: “You need to sell it like this; it is made in Australia, I say. It has had great success. It is translated into Norwegian and YES! You need to try this! I really say it like that” [Participant 1].

By “convincing”, GPs meant communicating with confidence, emphasizing aspects such as that they acknowledged the program as credible and that they had competence in MoodGYM because they had taken a course. They had positive experiences making the patient feel safe by suggesting strategies to ensure anonymity and noting that it was an evidence-based program. The availability and user-friendly nature of the program were also positive aspects mentioned by the GPs. They seemed to provide a rationale for MoodGYM when they recommended it, in the context of a trusting relationship with the patient.

After recommending MoodGYM, follow-ups were conducted in three different ways. One way was to recommend MoodGYM and not mention the program in further consultations. This way was explained as not being conscious about the intervention, and sometimes they did not record the recommendation making it impossible for them to remember to follow up: “But I haven’t, I just realized, I haven’t got any feedback from any of the patients…And I believe I haven’t written in the records that I have given the recommendation. So, I wouldn’t know to ask if they have tried it” [Participant 10].

A second way, described only by one GP (Participant 7) was to deploy the module follow-up manual in consultations in a structured motivating way. When the patients replied to questions about process issues, this GP noted that they told about episodes in life where principles from MoodGYM were applied. However, both the GP and the patient became impatient because time shortage and role conflicts were a problem. The module follow-ups revealed a struggle in the dialogue with patients because both the GP and patients wanted to explore problems from the patient’s everyday life as a starting point instead of discussing process issues related to MoodGYM. When problems in life were the starting point for the dialogue, neither the patients nor the GP were able to link it back to MoodGYM:

My role [with guided ICBT] was then in a way to motivate people and explore how it is going and…Like have you had…In the concrete program, right…Because first we were meant to talk about the modules and the things around that, but automatically we started talking about…It was totally unnatural for me not to ask: “How is it going at work?...She [the patient] was very interested in telling me…or wanted to talk about it [her mother and work] and…it was a very difficult role to have…and she became a little impatient…it didn’t work very well, that’s my opinion.

Participant 7

A third and most common way was to recommend MoodGYM but then limit the follow-up of the program to asking the patients if they had done it or not. Neither GPs nor the patients initiated talking about process issues apart from a few patients who gave brief feedback on whether they liked the program. GPs appreciated and encouraged the independent work undertaken by patients using MoodGYM. They admitted to not giving the program much attention in further consultations. As GPs made little effort to obtain feedback on MoodGYM from their patients, they also had little knowledge about how the patients perceived working at home with ICBT:

And to ask if they have gone into it [MoodGYM]. If it [depression] expanded in time…should they have sick leave? Have I done, not missed anything, right? And then…I ask about how it is going.

Participant 9

Interviewer: Do any of you…do you bring up or do you discuss anything from the program [MoodGYM] in your consultations?

No, I haven’t done it…I don’t get to know anything, but I just get to know if they liked it or not.

Participant 9

Despite GPs’ acknowledgement of the insufficiencies of standard treatment and regarding guided ICBT as a way to achieve improvement, paradoxically they returned to standard treatment instead of module follow-ups in subsequent consultations. Time constraints were also a challenge with standard treatment, although efforts to be flexible were made. Central to standard treatment was to support the patients telling their stories. GPs could recount these stories in detail in the interviews. Supportive tasks, such as being available for the patient, responding as a human being, and listening and acknowledging the patient’s problems were perceived to be most important in the treatment of depressed patients. These aspects of interaction were important in sustaining a trusting relationship and were part of standard treatment. No successful experiences to integrate process issues working with MoodGYM together with these supportive tasks were made explicit: “I guess it is [most important] to let the patient tell their story. It feels wrong to interrupt that type of story, about such things [when suffering from depression]. It is not so easy to cut to the chase—where in your stomach does it hurt?” [Participant 4] and “to strengthen the faith they might have in being able to become better. That there is hope. And…of course, to listen to them. To be willing to give more consultations and things like that” [Participant 2].

Confidence and viewing the patient as an active agent in treatment promoted GPs’ efforts to remember, find the time, and motivate patients to log on and work independently with ICBT. These efforts to recommend MoodGYM may relate to the NPT component of collective action or efforts to make an intervention doable [27,30]. Again, components of work to implement the module follow-ups were not successful or prioritized, and to accommodate constraints GPs either chose not to mention the program or briefly ask patients about adherence before returning to standard treatment in further consultations.

## Discussion

### Principal Findings

In this paper, we address aspects of a gap between the evidence from multiple trials that have found that ICBT can reduce the symptoms of depression (eg, [8-11]) and the lack of knowledge about implementing guided ICBT in the everyday clinical setting in general practice from the perspective of the GPs themselves. In the present study, a storyline was investigated starting from what prompted GPs to learn about ICBT, to how they applied the guided ICBT in their treatment of depression. Our findings imply that guided ICBT in practice was perceived to consist of two steps: (1) a consultation recommending use of MoodGYM and subsequently (2) module follow-up consultations. NPT suggests four main components of “work” to implement interventions: coherence (make sense), cognitive participation (engagement), collective action (efforts), and reflexive monitoring (feedback) [27,30]. In our study, the interplay between wanting to improve treatment, engaging by adding structured agenda, and empowering the patient—being efforts to the first step of recommendation—reflects three of the NPT components: coherence, cognitive participation, and collective action. Overall, GPs’ experiences with ICBT in our study demonstrated positive attitudes, as consistent with the literature [5,21,22]. On the other hand, the second step of module follow-ups was inhibited by various aspects, such as a hectic and varied practice, inadequate knowledge of the content of modules, and competing tasks of standard treatment and thus did not generate engagement. Mohr suggests the rationale of “what, why, when and how” must be defined in depth to enable development of implementable technical interventions to change behavior [33], and we argue that a health worker must be able to some extent answer these questions to integrate interventions into treatment. What module follow-ups should comprise, why they would improve treatment, and how they should be deployed was not made explicit by the GPs in our study. This incomplete rationale may contribute to the insufficient coherence and cognitive participation and might explain the little effort or little collective action in this part of treatment. NPT here contributes by providing a structure through which to understand how complex the challenges are that affect the GPs’ implementation of the module follow-ups.

A first step towards improvement was taken by GPs in our study because they made efforts to recommend MoodGYM; previous literature has found that GPs rarely recommend evidence-based self-management programs to patients with depressive symptoms [18,22]. An important aspect of promoting coherence and cognitive participation in MoodGYM was that it was based on CBT, a theory the GPs acknowledged as credible. Gunn and Palmer’s study emphasized that improved treatments for depression that are theory-based are more likely to promote cognitive participation and therefore be implemented [34]. Earlier findings show that GPs do not recommend online interventions because they lack faith in, and knowledge of, the content of the interventions [22]. Another study showed that GPs who know of trusted sites incorporate the Internet into their role as a GP [35]. In the present study, GPs engaged with MoodGYM and could convey information about the specific theory MoodGYM is based on, especially those who had taken the 3-day course. This indicated that knowledge about evidence and the theoretical base of an online intervention may promote use by GPs.

Competence was a recurring aspect that affected the GPs’ use of ICBT. Wanting to acquire competence promoted coherence, and experiencing competence gave the GPs’ work satisfaction and indicated cognitive participation. The GPs’ sense of competence was conveyed in encounters with patients, leading the GPs to make the effort to recommend the MoodGYM program, and it indicated collective action. The literature suggests that there is a relationship between the medical practitioner’s competence in the treatment of depression and patient outcomes [36,37]. Patients who receive guided ICBT also emphasize the importance of therapist’s competence [38]. The efforts or collective action GPs made to recommend MoodGYM to suitable patients in a motivating manner were often based on information they had learned at the course. Sinclair et al suggested redefining the role of the GPs to more of a consultant to enable proper use of ICBT during the treatment of depression [22]. Our findings suggest that with gained competence, GPs acted as consultants when they convincingly recommended MoodGYM and because they viewed their patients as active agents. This may indicate that it is consistent with the GPs’ role to recommend Internet self-help and that a course can enable GPs to gain the necessary competence and confidence to recommend ICBT to their patients.

In the present study, the second step was problematic; that is, the GPs were not successful in providing module follow-ups following initial recommendation of MoodGYM to their patients. Two important aspects were inadequate module knowledge and time shortages. It is possible that the course did not sufficiently emphasize the follow-ups to build competence for this part of treatment. Confidence could be enhanced by more practical training and highlighting the rationale for the module follow-ups (eg, that guided ICBT is more effective than self-administered [8,9,13]). It is well documented that organizational constraints, such as lack of time and a varied practice, can be unsupportive to implementing eHealth in general [39] and CBT or ICBT in general practice [40-42]. In our opinion, time is also flexible, and constraints may be facilitated with incitements.

Paradoxically, although GPs expressed the inadequacy of standard treatment, they still preferred this approach in follow-ups. Despite their enthusiasm for the structure of guided ICBT, GPs found it difficult to encourage patients to talk about MoodGYM process issues or to use process issues as a trigger to talk about everyday life concerns. NPT claims that efforts to implement an intervention occur within interactions with others [27,30]. Previous studies have shown that clinicians [22] and patients [43] prefer online interventions to be used as adjuncts rather than alternatives to standard treatment. Perhaps the patients’ expectations and preferences contributed to this choice in our study. An observational study from general practice found that when dealing with their patients’ mental health issues, GPs intuitively chose to listen to their patients’ problems contextualized in their everyday life, instead of being in control of the dialogue [44]. Both patients and GPs were more satisfied when the focus was on the patient as a whole person [34,44]. In our study, one GP (Participant 7) made explicit a role conflict she experienced between module follow-ups and her regular supporting role, indicating she saw the two types of care as dichotomous. Overall, our findings may imply that the focus on process issues working with modules is perceived as an instrumental approach, whereas when the depressed patients set the agenda by recounting their stories, a more patient-centered approach could be taken. This indicates a tension between the provisions of both structured therapeutic content of a program with a supportive patient-centered dialogue. Previous research from general practice has demonstrated this struggle between instrumental and patient-centered approaches [45], and the implementation of instrumental follow-ups does not fit with the GP’s role in depression treatment [46]. If GPs in our study had better knowledge of the modules and the rationale for module follow-ups and could link back to MoodGYM instead of having it as a starting point of the dialogue, coherence, cognitive participation, and efforts could be strengthened to enable implementation of module follow-ups combined with a patient-centred approach.

Given that the current evidence is not clear, it is not a surprise that GPs in our study lacked knowledge about the composition of support in guided ICBT. A review has suggested that, when studying depression, the effect size in symptom reduction appears to be greater for minimal-contact follow-ups where the patient is provided with a rationale (but no support focusing on process issues) for the use of self-help materials, compared with guided follow-ups with a focus on process issues [12]. In our study, guided ICBT was incomplete. On the other hand, the GPs made an effort to recommend and provide a rationale for their patients’ use of MoodGYM. The GPs also reported that they had offered follow-ups without process issues, during which they aimed to be available to their patients and to listen attentively. Such adjunct use to standard treatment may coincide with ICBT with minimal-contact follow-ups and perhaps is more compatible with the role of the GP.

### Strengths and Limitations

A methodological strength of this study was the use of in-depth interviews. The GPs had experienced these change processes and were thus able to elaborate on their experiences implementing ICBT. This study explored not only their attitudes but also the work involved in embedding the approach into regular practice. The second author (RSH) is a psychologist and was a therapist in a randomized controlled trial exploring guided ICBT and was a presenter of the course for the GPs in the present study, and the first author works as a GP. Both interviewers had prior understanding of ICBT from clinical settings. We hoped this would improve our understanding of the field and of the terminology; however, we tried not to presume that the GPs included in this study shared our perceptions [47]. RSH’s involvement in delivering the training might have led to a response bias if the GPs felt reluctant to be critical of the training or program. To reduce this possibility, we made it clear before each interview that we were not there to defend the treatment but that we wanted to better understand the GPs’ experiences. Another strength of the study was that the experienced third author (MBR) gave continuous feedback about our interviews to ensure sufficiently high quality and depth.

Although the sample size was appropriate for the needs of the study, we interviewed only 11 GPs from northern Norway, and our findings should be interpreted as only a partial description of the full range of GPs’ experiences. Participants were self-selected GPs enrolled in the course of the blended ICBT, and we cannot exclude the possibility of selection bias since participants may have been more interested in mental health or online interventions than the average GP. However, our main aim in this study was to explore aspects of the GPs’ experiences when they made the effort to implement ICBT in everyday practice and, accordingly, motivated GPs were acceptable. There was an overrepresentation of women at the course and therefore reflects participants interviewed. More male participants might have produced other stories, and a balanced gender analysis would have been possible. Only one GP (female) declined an interview invitation. NPT encourages exploring all stakeholders’ involvement [30], but time constraints and the necessary resources put this beyond the scope of this study.

### Conclusions and Implications

Perceptions of GPs in the present study contribute to our understanding of the challenges associated with changing the treatment of depression in general practice. A need to supplement standard treatment was prominent, and GPs endorsed the principles of guided ICBT. Guided ICBT was seen as involving two steps. The first step was to recommend MoodGYM and efforts were made to integrate this step into treatment. This indicates that recommending ICBT can add a valuable tool to GP’s toolkit. Offering training and highlighting the following aspects may increase recommendation of ICBT by GPs: (1) ICBT is theory-based and credible, (2) ICBT increases GPs’ work satisfaction having a tool to offer, and (3) ICBT facilitates empowerment of patients in their own health.

The second step was to integrate module follow-ups into treatment. GPs expressed that they had difficulties with this step and instead returned to standard treatment. A number of reasons and paradoxes were identified when exploring this incomplete implementation. Our study indicates that recommending a theory-based Internet self-help program is acceptable within the role of a GP, however, unclear for module follow-ups. More practical training and providing incentives to enable GPs to prioritize time to complete the online program themselves to obtain knowledge may improve inadequate knowledge of the modules. Our findings imply that it is important to have a patient-centered approach in the follow-ups. More research is needed to explore what the support of ICBT should comprise when deployed in the context of general practice. A key question is to investigate if GPs can combine patient-centered follow-ups with process issues, or if adjunct use of ICBT to standard treatment is more suitable.

## Multimedia Appendix 1

Interview guide.

## Abbreviations

CBT: cognitive behavioral therapy
GP: general practitioner
ICBT: Internet-based cognitive behavioral therapy
NPT: normalization process theory
