# Supplementary material for: Norwegian General Practitioners’ Perspectives on Implementation of a Guided Web-Based Cognitive Behavioral Therapy for Depression: A Qualitative Study
Source: J Med Internet Res. 2014 Sep 10;16(9):e208. doi: 10.2196/jmir.3556 (PMC4180343; doi:10.2196/jmir.3556)
Supplement: Supplementary file 1 [file jmir_v16i9e208_app1.pdf]

Interview guide

-date and place
-age and experience from general practice

General perceptions

-Tell a little bit about your experience with depressed patients and their treatment.
-How do you experience having a depressed patient in your office?
-In what way do you approach this patient?
-Tell about the last depressed person you had in your office (including contact, actions…)

Motivation

-What made you take the course in CBT? How did you hear about MoodGYM?
-Describe how you have tried to conduct treatment.
-If anything, what motivated you as a doctor using techniques of CBT or MoodGYM?
-What elements of treatment do you think are most important? How does this affect treatment progress?
-What difficulties have you experienced and how did you solve them?

 -Did the patients want to try this?
 -Did the patients do the modules at home?
 -Were there any technical challenges?
 -Was there enough time in the consultation?
 -How does it fit in a normal consultation? Tell more!-What is necessary for you to prioritize to obtain knowledge/experience about MoodGYM?
 -What is necessary for you to feel well prepared to use MoodGYM?

Treatment

-What do you think about using CBT or MoodGYM?
-How did you as a doctor perceive using MoodGYM?
- Who do you think can be a candidate for this treatment? (What is it about the patient that makes him suitable or not?)
-Approximately how many patients have you tried this on?
-Tell about a situation you remember well from this time you have tried to use MoodGYM in your treatment. Why this episode?
-Did you have to do any practical changes in consultations to enable treatment? (Time, follow-ups, other?)
-Did you do any preparation before using MoodGYM?
-Did you experience changes in the contact/bond with the patient? Tell about the last one.
-Did you experience you could influence the approach? In what way?
-Did you make any adjustments to the approach to accommodate how you preferred to meet your patients?
-Can you tell what you valued from this way of providing treatment?

-If you were to recommend this to a colleague what would you emphasize?
-With the experiences you have made, if you had the opportunity to improve MoodGYM what would you change, take away or add?
-What other treatment alternatives did you consider?

Quality and interaction.

-Has this course based on CBT resulted in a change treatment for your patients? Describe.
-Has your use of MoodGYM changed the way you give treatment?
-How do you perceive your cooperation with the specialized service after you have taken this course?
-Did you change how you interact with specialized service after you implemented MoodGYM? How?
-Would you like to take another course based on CBT and perhaps learn more about MoodGYM or another self-help program? Elaborate.

Final questions

Would you like to add anything?
Anything I haven't asked, that you would like to elaborate?

Thank you!
